# P2 Receptor Antagonists Rescue Defective Heme Content in an In Vitro SLC25A38-Associated Congenital Sideroblastic Anemia Cell Model

**DOI:** 10.3390/ijms252413314

**Published:** 2024-12-12

**Authors:** Antonella Santoro, Silvia De Santis, Ferdinando Palmieri, Angelo Vozza, Gennaro Agrimi, Immacolata Andolfo, Roberta Russo, Antonio Palazzo, Clelia Tiziana Storlazzi, Arianna Ferrucci, Yong Woong Jun, Eric T. Kool, Giuseppe Fiermonte, Achille Iolascon, Eleonora Paradies, Carlo Marya Thomas Marobbio, Luigi Palmieri

**Affiliations:** 1 Department of Biosciences, Biotechnology and Environment, University of Bari Aldo Moro, 70125 Bari, Italy; antonella.santoro@uniba.it (A.S.); silvia.desantis@uniba.it (S.D.S.); ferdinando.palmieri@uniba.it (F.P.); angelo.vozza@uniba.it (A.V.); gennaro.agrimi@uniba.it (G.A.); antonio.palazzo@uniba.it (A.P.); cleliatiziana.storlazzi@uniba.it (C.T.S.); giuseppe.fiermonte@uniba.it (G.F.); luigi.palmieri@uniba.it (L.P.); 2 CNR Institute of Biomembranes, Bioenergetics and Molecular Biotechnologies (IBIOM), 70126 Bari, Italy; arianna.ferrucci.bio@gmail.com; 3 Center of Excellence in Comparative Genomics, University of Bari Aldo Moro, 70125 Bari, Italy; 4 Department of Molecular Medicine and Medical Biotechnologies, University of Naples Federico II, 80131 Naples, Italy; immacolata.andolfo@unina.it (I.A.); roberta.russo@unina.it (R.R.); achille.iolascon@unina.it (A.I.); 5 CEINGE-Biotecnologie Avanzate Franco Salvatore, 80145 Naples, Italy; 6 Department of Chemistry, Korea Advanced Institute Science and Technology (KAIST), Daejeon 34141, Republic of Korea; ywjun@kaist.ac.kr; 7 Department of Chemistry, Stanford University, Stanford, CA 94305, USA; kool@stanford.edu; 8 Sarafan ChEM-H Institute, Stanford University, Stanford, CA 94305, USA

**Keywords:** congenital sideroblastic anemia (CSA), SLC25A38, mitochondrial carriers, pyridoxal 5′-phosphate, P2 receptors, heme biosynthesis, oxidative stress, iron dyshomeostasis

## Abstract

Mutations in the SLC25A38 gene are responsible for the second most common form of congenital sideroblastic anemia (CSA), a severe condition for which no effective treatment exists. We developed and characterized a K562 erythroleukemia cell line with markedly reduced expression of the SLC25A38 protein (A38-low cells). This model successfully recapitulated the main features of CSA, including reduced heme content and mitochondrial respiration, increase in mitochondrial iron, ROS levels and sensitivity to oxidative stress. Notably, our study uncovered a new role for extracellular pyridoxal 5′-phosphate (PLP) and other P2 receptor antagonists in rescuing the altered parameters of A38-low cells (for example, the heme content of the A38-low cells was increased from about 50% to about 80% by the P2 receptor antagonists treatment compared with the value of the controls). These findings suggest that targeting P2 receptors could represent a promising therapeutic approach for SLC25A38-associated CSA.

## 1. Introduction

Congenital sideroblastic anemia (CSA) is a syndrome comprising a group of rare and heterogeneous diseases related to ineffective erythropoiesis and secondary iron overload. It is caused by mutations in genes involved in heme biosynthesis, mitochondrial protein synthesis, iron-sulfur (Fe-S) cluster biogenesis, or Fe-S cluster transport [1]. CSA is characterized by the presence of ringed sideroblasts in the bone marrow, which are nucleated erythroblasts exhibiting an abnormal accumulation of non-heme iron granules within the mitochondrial matrix [2]. Various forms of CSA have been identified at the molecular level, providing insights into cellular pathways linked to dysfunctional iron metabolism within the mitochondria [3]. The most prevalent form of CSA is X-linked sideroblastic anemia (OMIM #300751), which is caused by a deficiency in δ-aminolevulinic acid synthase 2 (ALAS2) [4], a pyridoxal 5′-phosphate (PLP)-dependent enzyme that catalyzes the synthesis of δ-aminolevulinic acid (δ-ALA) within the erythroid cells [5,6]. Vitamin B6 treatment, i.e., pyridoxine treatment, which enhances intracellular PLP levels, sometimes yields positive responses in X-linked CSA [7]. The second most common form of CSA (OMIM #205950) is caused by mutations in the SLC25A38 gene (CSA-SLC25A38) [8], which encodes a member of the mitochondrial carrier family [9]. The function of SLC25A38 was first elucidated in 2009, when Guernsey et al. provided the initial evidence linking this transporter to heme biosynthesis [10]. In a subsequent study, Lunetti et al. characterized the SLC25A38 protein as a mitochondrial glycine carrier [11]. The importance of this gene was further validated through zebrafish [12] and mouse [13] models, both demonstrating the crucial function of this gene in erythropoiesis. So far, despite the availability of these models, no therapy exists for the treatment of CSA-SLC25A38. Bone marrow transplantation has shown limited success in only a few cases [14]. Furthermore, patients affected by CSA-SLC25A38 do not respond to vitamin B6 treatment nor to folic acid and glycine [8,15]. Recently, a relationship of SLC25A38 with purine metabolism was observed [16]. In particular, SLC25A38, together with the mitochondrial aspartate–glutamate carrier [9], are located in close proximity to the purinosome in order to facilitate the channeling of glycine and aspartate, which are needed for purine synthesis [16].

In this study, in order to examine possible therapeutic strategies, we generated an erythroleukemic K562 cell line with markedly reduced expression of the SLC25A38 protein (A38-low), which effectively recapitulates the main features of CSA-SLC25A38. In particular, we observed that the cellular defects were rescued by the addition of PLP and P2 receptor antagonists in the growth medium, paving the way for novel potential therapies of CSA-SLC25A38.

## 2. Results

### 2.1. Phenotypic Characterization of the A38-Low Cell Line

**Figure 1:**
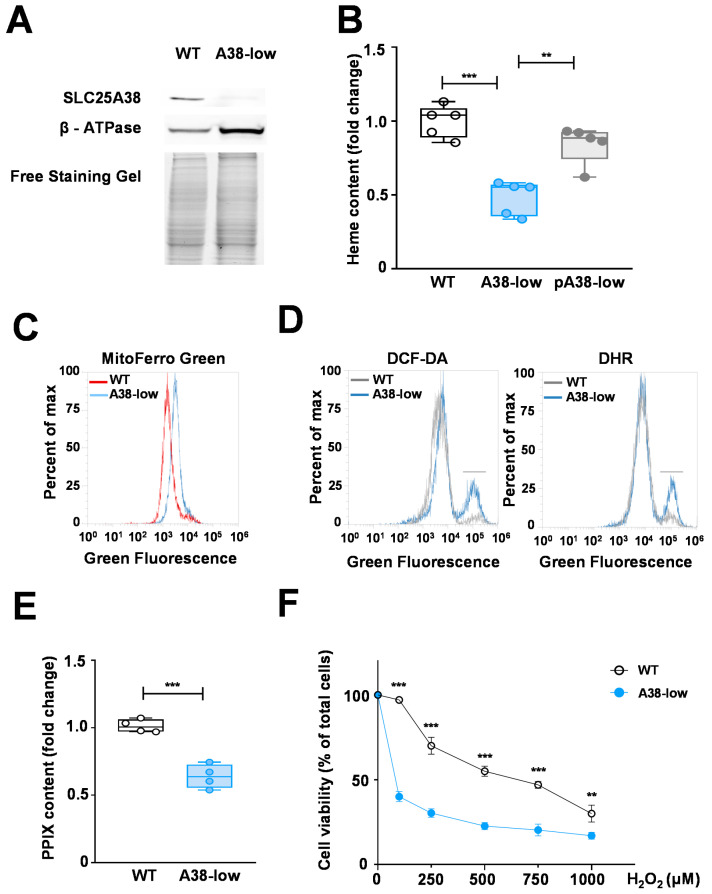
(**A**) Expression level of SLC25A38 in A38-low and K562 WT cell lines. Total cellular proteins (50 µg) from the indicated cells were probed by a Western blot analysis for SLC25A38 expression. β-ATP synthase and free staining gel served as loading control. (**B**) Heme content in K562 cell lines. Heme content was determined in K562 cell lines from wild type (WT) (white), A38-low (blue) and A38-low cells transfected with pcDNA3.1-SLC25A38 plasmid (pA38-low) (grey) (** *p* < 0.01, *** *p* < 0.001, Student’s *t* test; *n* = 5). (**C**) Mitochondrial LIP in K562 cell lines. A38-low and WT cells were stained with 5 µM Mito-FerroGreen and analyzed using an Attune Acoustic Focusing Cytometer. (**D**) ROS levels in K562 WT and A38-low cells. Representative histograms illustrating the cellular and mitochondrial ROS levels were determined by cytofluorimetric analysis using 2′,7′-dichlorodihydrofluorescein diacetate (DCF-DA) and dihydrorhodamine 123 (DHR), respectively. The short black line in the figure highlights the populations with elevated oxidative stress. (**E**) PPIX contents in K562 WT (white) and A38-low cells (blue). PPIX was determined in total cellular extracts by a fluorescence method [21]. The fluorescence values were normalized to mg/mL of each sample. PPIX content is expressed as the control-related fold change (*** *p* < 0.001, Student’s *t* test; *n* = 4). (**F**) Oxidative stress sensitivity. Cell viability of indicated cells, assessed through the resazurin assay, was determined following a 48 h treatment with H_2_O_2_ at the indicated concentrations. The data are presented as a percentage of the control group (untreated), (** *p* < 0.01; *** *p* < 0.001, two-way ANOVA test; *n* = 3).

We generated a K562 erythroleukemia cell line knocked down for SLC25A38 (A38-low) by using CRISPR/Cas9 technology, which displayed a heterozygous deletion of 11 nucleotides. This deletion resulted in a frameshift mutation after Gln13, producing a truncated peptide (Supplementary Figure S1). The content of SLC25A38 in A38-low cells was markedly reduced (about 25% of the WT cell content), as confirmed by Western blotting (Figure 1A).

**Figure 2:**
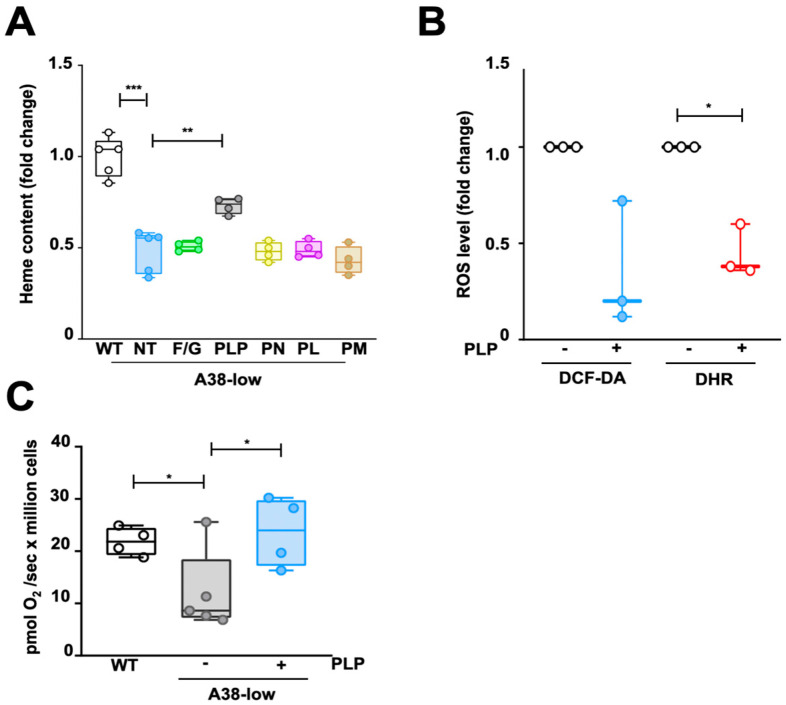
Effect of folic acid with glycine and B6 vitamers on A38-low cells. (**A**) Heme content, assessed in K562 WT, as well as in the A38-low cell line untreated (NT) or treated for 48 h with a mixture of 1 mM folic acid and 100 mM glycine (F/G), or with pyridoxal 5′-phosphate (PLP), pyridoxine (PN), pyridoxal (PL), and pyridoxamine (PM) at the concentrations of 0.1 mM. The values are expressed as the fold change related to the control cells. Number of replicates: 4 or 5. (**B**) Cellular and mitochondrial ROS levels in A38-low cells untreated (−) or treated with 0.1 mM PLP (+), expressed as the fold change in median fluorescence intensity as determined by cytofluorimetric analysis, using 2′,7′-dichlorodihydrofluorescein diacetate (DCF-DA) and dihydrorhodamine 123 (DHR), respectively. Number of replicates: 3. (**C**) State III respiration, in the presence of malate, pyruvate, glutamate, succinate and ADP, assessed in WT and in A38-low cell lines. Additionally, A38-low cells were treated with 0.1 mM PLP for 48 h. Comparative measurements were conducted in a pairwise manner against their respective control counterpart (* *p* < 0.05; ** *p* < 0.01; *** *p* < 0.001; Student’s *t*-test; *n* = 4 or 5).

As a control, by loading the same amount of cellular protein, no significant variation in protein content between A38-low and WT cells was observed (Figure 1A). Mutant cells were phenotypically characterized to verify the presence of defects typical of CSA. Initially, we explored whether the cellular model exhibited a defect in heme content using a colorimetric assay [12] (Figure 1B). A38-low cells showed a reduction in cellular heme content compared with WT cells, equal to 47.4 ± 12.7%, which was rescued by the plasmid expression of the WT SLC25A38 gene (Figure 1B). Then, we measured the mitochondrial iron content by employing Mito-FerroGreen, a specific fluorescent probe capable of detecting the mitochondrial labile iron pool (mLIP) [17]. Our findings revealed an elevated mLIP in the A38-low cell line compared with the wild-type counterpart (Figure 1C). Given that the mitochondrial iron increase is related to oxidative stress [18], we also assessed cellular and mitochondrial ROS levels by flow cytometry analysis using DCF-DA and DHR fluorescent probes, respectively. A38-low cells exhibited an increase in both cellular and mitochondrial ROS levels compared with WT cells (Figure 1D). Furthermore, using a high-resolution respirometer [19,20], a significantly diminished state III respiration was observed in A38-low cells, indicating a decline in the respiration performance exhibited by the mutant cells (Figure 2C). In addition, to confirm that the reduction in heme content directly depended on the lack of heme precursors and not on secondary effects, i.e., an increase in mLIP and/or ROS production, we determined the cellular content of PPIX, that is, the last precursor for heme biosynthesis before iron incorporation [6]. As indicated in Figure 1E, a reduction of 37.6 ± 7.5% in PPIX levels in A38-low cells compared with the WT control was observed, in agreement with the known role of SLC25A38 in heme synthesis [11]. Finally, in order to further characterize our cell model, we studied the cell viability upon hydrogen peroxide treatment. As shown in Figure 1F, the A38-low cell line showed a higher sensitivity to oxidative stress with respect to the WT cells. In fact, the treatment with 100 µM hydrogen peroxide resulted in an approximately 60% decrease in the cell viability of the A38-low cell lines while having no effect on WT cells.

### 2.2. Impact of Folic Acid, Glycine, and B6 Vitamers on the Phenotypic Defects of A38-Low Cell Line

In a next step, we tested different compounds for their potential ability to alleviate the phenotype in A38-low cells. We chose folic acid plus glycine because they have been reported to exert beneficial effects on the zebrafish model [12]. We further investigated the effects of B6 vitamers, including pyridoxine (PN), pyridoxal (PL), pyridoxamine (PM), and pyridoxal 5′-phosphate (PLP), as recent studies have revealed an unexpected conditional synthetic lethality between the heme synthesis and pyridoxine deficiency in a mouse model of CSA-SLC25A38 [13]. The synergistic treatment of folic acid plus glycine determined no significant effect on the A38-low cell line (Figure 2A).

Surprisingly, only PLP, but not the others B6 vitamers, was able to restore the heme content in A38-low cells (Figure 2A). A concentration of 0.1 mM PLP in the culture medium for a duration of 48 h exhibited the most significant rescue effect, leading to a 30 ± 8% increase in heme content and effectively reaching levels comparable with those of the wild-type cells (Figure 2A and Figure S2). As a control, the addition of the tested substances to the culture medium did not induce any variation in cell viability (Supplementary Figure S3) and did not result in any significant variation in the heme content in the control cells. Finally, we tested whether the beneficial effect of PLP extended to other A38-low specific defects (see above). In this regard, PLP treatment markedly reduced cellular and mitochondrial ROS levels (Figure 2B) and completely restored mitochondrial respiration in A38-low cells (Figure 2C), suggesting an ameliorative effect on the secondary effects of SLC25A38 deficiency as well.

### 2.3. Decrease in PLP Content in A38-Low Cells and Its Restoration by Vitamin B6 Derivatives

**Figure 3:**
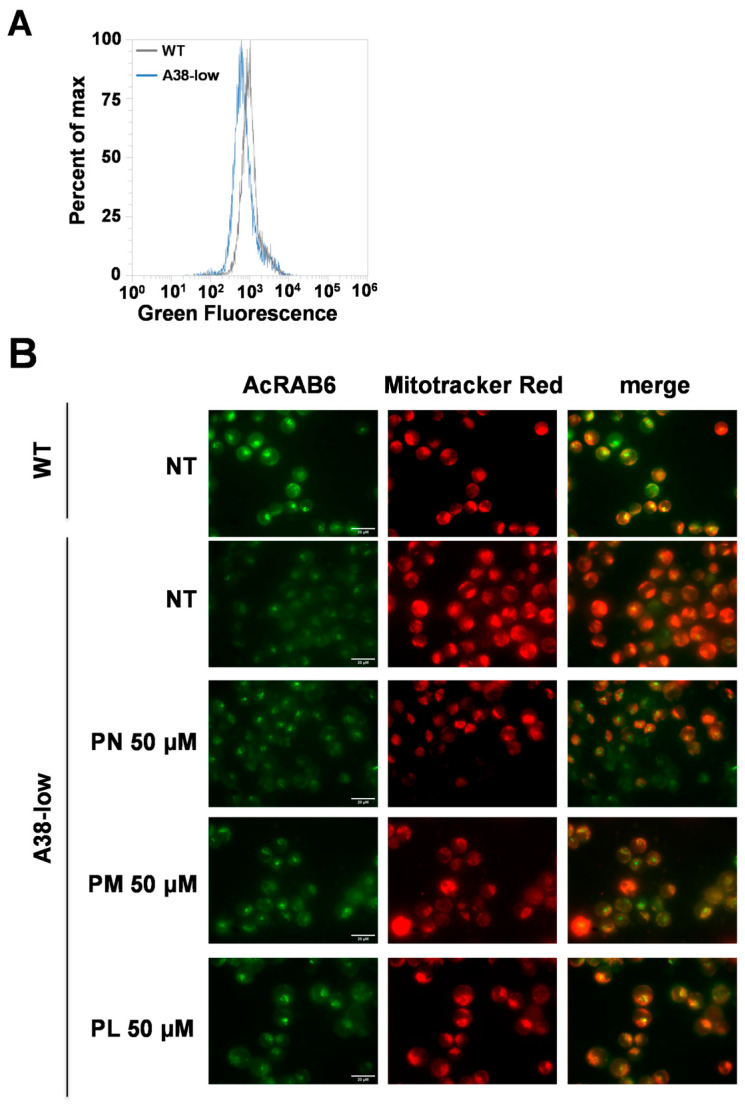
Pyridoxal 5′-phosphate content in WT and A38-low cell lines. (**A**) Representative histograms illustrating the intracellular pyridoxal 5′-phosphate (PLP) content, determined by cytofluorimetric analysis using the AcRAB6 probe, were obtained for the K562 WT and A38-low cell lines. (**B**) Epifluorescence images showing untreated K562 WT and A38-low cells (NT), along with A38-low cells treated with 50 µM pyridoxine (PN), pyridoxamine (PM), and pyridoxal (PL), and stained with AcRAB6 for 12 h as reported [22].

The unexpected result of the PLP effect on A38-low cells might suggest a potential decrease in intracellular PLP content in these cells. Figure 3A shows PLP levels in A38-low and control cells determined by flow cytometry with a coenzyme-specific fluorescent probe (AcRAB6) [22].

Indeed, A38-low cells (Figure 3A) showed a reduction in intracellular PLP levels compared with the control. It is known that PN, PL, and PM are able to enter cells and then be converted into PLP [23]. Therefore, our finding that PLP, but not its precursors, is able to rescue the phenotype of our cell model could suggest an apparent defect in the cellular acquisition of precursors and/or in their conversion to PLP. To test this hypothesis, we evaluated the levels of intracellular PLP in A38-low cells after treatment with PN, PL, and PM. As reported in Figure 3B, after these treatments, the PLP content was increased in A38-low cells. These outcomes indicate that the ameliorative effect of PLP on cellular models is not related to the restoration of intracellular PLP content.

### 2.4. Effect of P2 Receptor Antagonists on WT and A38-Low Cell Lines

**Figure 4:**
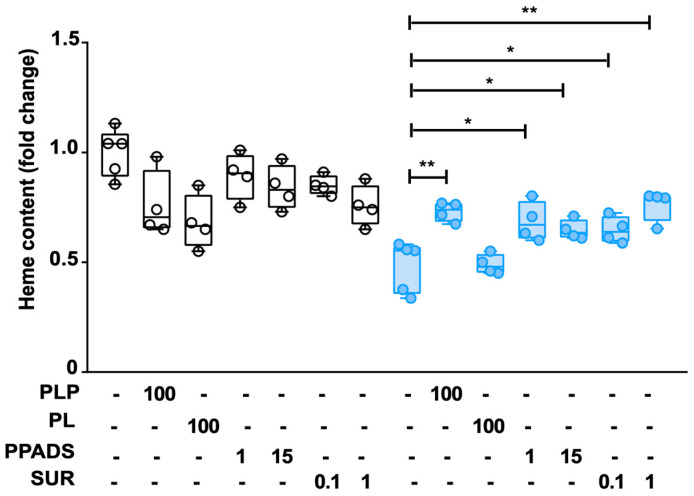
Involvement of the P2 receptor in the PLP-mediated effect. Heme content was determined in total cell extracts from K562 WT (white circle) and A38-low cells (blue circle) after treatment with pyridoxal 5′-phosphate (PLP), pyridoxal (PL), pyridoxalphosphate-6-azophenyl-2′,4′-disulfonic acid (PPADS), and suramin (SUR) at the indicated concentrations (µM) for 48 h. Data were indicated as the control-related ratio (* *p* < 0.05; ** *p* < 0.01; Student’s *t*-test untreated-related; *n* = 4 or 5).

The inability of the B6 vitamers PN, PL, and PM to rescue the defect of heme content in A38-low cells indicated a distinct role of PLP in this process. Notably, extracellular phosphorylated B6 vitamers are unable to penetrate cell membranes and must undergo conversion to non-phosphorylated forms by external phosphatases before cellular uptake [23]. In other words, in the presence of external phosphatases, PLP added to the cell culture medium may be transported across the cell membrane as PL and then converted back to PLP by intracellular pyridoxal kinase. This information, together with the inability of non-phosphorylated B6 vitamers to rescue the heme content defect in the A38-low cell line, suggest that the effects of PLP in these cells are primarily exerted extracellularly. Interestingly, PLP has already been shown to act as an effective antagonist of purinergic P2 receptors [24,25,26], which are cell membrane receptors playing pivotal roles in numerous physiological processes [27,28]. To evaluate whether the restorative effect of PLP is dependent on purinergic P2 receptors, we investigated the impact of pyridoxal phosphate-6-azophenyl-2′-4′-disulfonic acid (PPADS), a synthetic PLP analogue known for its P2 receptor antagonist properties [29]. Additionally, we tested the effect of suramin, which is another P2 receptor antagonist, with a structure unrelated to PLP [29]. Interestingly, both PPADS and suramin restored the heme content in A38-low cells as PLP, while PL was ineffective (Figure 4). Furthermore, the treatments with PPADS and suramin did not exert any change in heme content in the WT cell (Figure 4), or any changes in cell viability respect to untreated cells (Supplementary Figure S4).

## 3. Discussion

The second most common form of CSA is caused by mutations in the SLC25A38 gene, encoding a transporter [9] that provides glycine to δ-aminolevulinic synthase, the first enzyme of heme biosynthesis localized within the mitochondria and containing pyridoxal 5′-phosphate (PLP) as a cofactor [5,6]. To elucidate the molecular mechanisms of CSA-SLC25A38 and develop a potential therapeutic strategy for this disease, two models, zebrafish and mouse models, have been produced [12,13]. Specifically, in the zebrafish model, supplementation with a combination of glycine and folate successfully restored the heme content defect [12], and in the mouse model, a pyridoxine-deficient diet dramatically worsened the hematological parameters, suggesting a potential therapeutic role for vitamin B6 [13]. However, the treatments in animal models with either the combination of glycine and folate or vitamin B6 showed any beneficial effects in patients affected by CSA-SLC25A38 [8,15] as well as in our cell model, as specified below.

Until now, no human cell lines have been tested as a model for studying CSA-SLC25A38. Although the in vitro cell models lack the complexity of the in vivo systems, such as the interactions within the bone marrow microenvironment and the dynamics of systemic pharmacodynamics, they have been proven to be valuable in the study of CSA caused by mutations in HSCB [30], ABCB7 [31], ALAS2 [32], and GLRX5 [33] genes. In this study, we have characterized for the first time a human cellular model for SLC25A38-associated CSA, named A38-low cells, exhibiting the main defects distinctive of SLC25A38 deficiency [reduction of heme content (Figure 2A) and mitochondrial respiration (Figure 2C), and increase in mitochondrial iron content (Figure 1C) and cellular and mitochondrial ROS (Figure 1D) as well as greater sensitivity to oxidative stress (Figure 1E)]. This in vitro cell model has been tested for the ability of the aforementioned compounds to rescue the cellular defects characteristic of CSA-SLC25A38. The results presented in this study demonstrate that (i) treatment with folate and glycine has no positive effect on the A38-low cell line (Figure 2A), unlike the results obtained in the zebrafish model [12], but were consistent with what was observed in patients [15]; (ii) the B6 vitamers PN, PL, and PM do not exert a compensatory effect on the intracellular heme content of the mutant cells (Figure 2A), again in agreement with the results obtained in patients [8] and in contradiction with our expectations based on the suggestion of a potential therapeutic role for vitamin B6 [13]; and (iii) PN, PL, and PM are able to increase the intracellular PLP, which is reduced in A38-low cells (Figure 3A,B). In contrast, surprisingly, exogenous PLP rescues the defects in heme content, ROS production, and state III respiration in A38-low cells (Figure 2A–C).

Our data strongly suggest that the beneficial effects of the PLP treatment are exerted outside the cells through a receptor-mediated biosignaling pathway. Indeed, PLP, unlike the other B6 vitamers, is able to act at the cell surface as an antagonist of purinergic receptors [24,25,26], known as P2 receptors [27,28]. Furthermore, this interpretation is supported by our finding that PPADS and suramin, two known P2 receptor antagonists [29], are able to reverse the defect in heme content in our CSA-SLC25A38 cell model (Figure 4), as well as our observation that PN, PL, and PM increase the intracellular PLP content without affecting the A38-low cell heme content. Notably, P2 receptors are present in erythroid cells [34], although their involvement in hematopoiesis has not been elucidated yet. On this basis, we hypothesize that activation of P2 receptors in A38-low cells disrupts cell signaling and impairs heme biosynthesis. Interestingly, among the substances used as antagonists of P2 receptors in this work, a therapeutic effect for PLP and suramin in various pathologies has been reported [35,36,37,38,39]. We propose that the lack of response to vitamin B6 in patients, which is transformed in PLP in liver [40], is caused by the inability of PLP to reach a sufficient concentration on the surface of erythroid cells due to the action of extracellular phosphatases [23]. We further propose that P2 receptor antagonists resistant to phosphatases could represent a promising therapeutic approach for SLC25A38-associated CSA. In future work, in order to enhance the understanding of the SC25A38-related CSA and the development of effective treatment, it will be necessary to (i) validate in vivo the therapeutic potential and safety of the P2 receptor antagonists by using animal models or clinical trials, (ii) understand the molecular mechanisms underlying the effects of the P2 receptor antagonists in rescuing heme content and other cellular defects of the SLC25A38-low cell model, (iii) assess the long-term implications of using P2 receptor antagonists, and (iv) compare the efficacy of these compounds with other potential treatments for SLC25A38-associated CSA.

## 4. Materials and Methods

### 4.1. Reagents, Cell Lines, and Culture Conditions

All chemicals and reagents used in this study were obtained from Sigma Aldrich (St. Louis, MO, USA), except for dihydrorhodamine 123 (DHR, cat. D23806) and 2′,7′-dichlorodihydrofluorescein diacetate (DCF-DA, cat. D399), which were purchased from Molecular Probe (Thermo Fisher Scientific, Waltham, MA, USA). The cell culture medium was obtained from Euroclone, (Pero, Milan, Italy). K562 human erythroleukemic cell lines (ATCC, Manassas, VA, USA, Cat# CCL243) were cultured in Roswell Park Memorial Institute medium (RPMI 1640) supplemented with 2 mM glutamine, 16% fetal bovine serum, 100 U/mL penicillin, and 0.1 mg/mL streptomycin [41]. All cell lines were maintained at 37 °C in a humidified atmosphere with 5% CO_2_ in 25 cm^2^ culture flasks positioned upright. The cell concentrations were adjusted to 5 × 10^5^ cells/mL for K562 to ensure an exponential growth phase. Fresh medium was provided every 2–3 days to sustain cell growth. During compound assays, antibiotics were excluded. Cells were treated with drugs and vitamers at the specified concentrations for 2 or 3 days. The medium containing the reagents was changed daily during the treatment period.

### 4.2. CRISPR/Cas9 Editing of the SLC25A38 Gene

Mutagenesis of K562 cells was performed using Lonza nucleofection technology, following the optimized protocols provided by the manufacturer. In brief, cells were gently suspended in a total volume of 100 µL of SF Cell Line 4D-Nucleofector Solution. Then, 1 µg of gRNA (CB-9571.2 IVT gRNA) (Supplementary Table S1) and 1 µg of Cas9 protein (TrueCut Cas9 protein, Thermo Fisher Scientific, Waltham, MA, USA) were added to the cell suspension. The mixture was pulsed using the FF-120 program. Immediately after nucleofection, the cells were transferred into a pre-warmed fresh medium in six-well plates. After 48 h, a portion of the cells was analyzed for in situ mutagenesis using the GeneArt Genomic Cleavage Detection Kit (Thermo Fisher Scientific, Waltham, MA, USA). Subsequently, a serial dilution was performed to obtain cell clones. Each clone was subjected to sequencing using two primers (CB-9571.2_GCD1F/R) located upstream and downstream of the mutation site of interest (Supplementary Table S1).

### 4.3. SLC25A38 Gene Expression in A38-Low Cells

The coding sequence for SLC25A38 (Accession No. NM_017875) was amplified by PCR from Jurkat cells’ cDNA using specific forward and reverse oligonucleotide primers corresponding to the extremities of the coding sequence, flanked by *Hind*III and *Eco*RI restriction sites, respectively. Subsequently, the amplified sequence was cloned into the pcDNA3.1(+) expression plasmid (Invitrogen, Thermo Fisher Scientific, Waltham, MA, USA), and the construct was transformed into E. coli TG1 cells (Invitrogen). Transformants selected on LB (10 g/L tryptone, 5 g/L yeast extract, 5 g/L NaCl, pH 7.4) plates containing 100 µg/mL ampicillin were screened by direct colony PCR and verified by sequencing the inserts.

The pcDNA3.1-SLC25A38 plasmid was transiently transfected into A38-low cells by nucleofection using 1 µg of plasmid and the above-mentioned parameters. For the selection, transfected cells were grown with 500 µg/mL of G418 (Thermo Fisher Scientific, Waltham, MA, USA) for 72 h. The surviving cells were harvested, and SLC25A38 protein expression was checked by a Western blot analysis.

### 4.4. Western Blot Analysis

Cells were washed three times and then lysed using a cell lysis buffer containing 0.15 M NaCl, 5 mM EDTA, 1% NP-40, and 10 mM Tris-Cl at pH 7.4. The lysate was transferred to a microcentrifuge tube, and the protein concentration was determined using the Bradford protein assay reagent from Bio-rad (Hercules, CA, USA). Subsequently, 50 µg of cell proteins were separated on 12% polyacrylamide gels [42] and transferred onto nitrocellulose membranes (Bio-rad, Hercules, CA, USA) using an electroblotting technique [43]. The membranes were washed for 20 min with TBST buffer and then incubated with primary antibodies, including anti-SLC25A38 (Thermo Fisher Scientific, Waltham, MA, USA, cat. PA5-42472 1:500) and anti-βATPase (BD Bioscience, Milan, Italy, cat. 612518, 1:10,000). The anti-SLC25A38 and anti-βATPase antibodies were incubated for 1 h at RT. After three washes with TBS for 15 min each, the membranes were incubated with secondary antibodies, anti-mouse (Pierce, Thermo Fisher Scientific, cat. 31430, 1:10,000) and anti-rabbit (Pierce, Thermo Fisher Scientific, cat. 31402, 1:10,000), for 1 h at room temperature. Finally, the membranes were washed three times with TBS, and the immunoreactive bands were visualized using Immobilon Western Chemiluminescent HRP Substrate from Millipore (Merck KGaA, Darmstadt, Germany) as described previously [44].

### 4.5. Cytotoxicity Assay

To assess cytotoxicity, cells were seeded into 96-well plates at a density of 2 × 10^4^ cells per well in 0.1 mL of RPMI, treated with different compounds at the indicated concentrations for 48 h. Then, cell viability was analyzed using the resazurin assay, as reported by the manufacturer (AlamarBlue, Bio-Rad, Hercules, CA, USA, cat. BUF012A). Briefly, the medium was replaced with a working solution (10% resazurin in medium), and the plates were incubated for 4 h after dye addition. In order to calculate the percent reduction of resazurin by viable cells, absorbance was measured in triplicate for each point at 570 and 600 nm using a microplate spectrophotometer (Victor 3, Perkin Elmer, Milan, Italy). Cell viability was determined by comparing the absorbance of treated cells with untreated controls [45].

### 4.6. Protoporphyrin IX and Heme Content Assays

For PPIX synthesis measurement, the method described by Novak and colleagues was followed [21]. All conditions were analyzed in triplicates. In brief, 2 × 10^5^ cells per well were treated with 20 µL of lysis buffer (comprising 125 mM Tris/HCl, pH 7.8; 2 mM DTT; 2 mM EDTA; 10% (*v*/*v*) glycerol; 1% (*v*/*v*) Triton X-100; all from Sigma Aldrich (St. Louis, MO, USA) and incubated for 30 min with gentle shaking. Cell lysis was visually confirmed under a microscope. Once complete lysis was achieved, 80 µL of PPIX extraction solution (consisting of 50% methanol, 35.7% distilled water, 14.3% perchloric acid pre-dissolved to 70% in water, all *v*/*v*) was added, and the cells were shaken for an additional 45 min. Simultaneously, the protein content of the lysed samples was determined using the Bradford assay (Bio-Rad, Hercules, CA, USA). Subsequently, cell extracts were transferred to a black-walled 96-well plate for PPIX fluorescence measurement. The buffer used was previously confirmed not to interfere with PPIX fluorescence measurements. PPIX fluorescence was assessed using a Victor Multilabel Plate Reader (Perkin Elmer, Milan, Italy) with a 405 nm excitation and a 620 nm emission filter, with an integrated readout period of 10 sec per well. Background fluorescence was subtracted by measuring a mixture of 20 µL of lysis buffer and 80 µL of extraction buffer. The obtained data were normalized to the protein content (mg) per sample and presented as relative PPIX content units (arbitrary units). For the quantification of heme, a commercial hemin (oxidized heme) colorimetric assay from Sigma Aldrich (St. Louis, MO, USA; cat. MAK036) was utilized, following the manufacturer’s guidelines and as previously published [12]. All samples were analyzed in triplicates. Initially, cells were homogenized rapidly in 4 volumes of cold hemin assay buffer, followed by centrifugation at 13,000× *g* for 10 min at 4 °C to remove insoluble material. The resulting supernatant was diluted 1000-fold with hemin assay buffer. The reaction mix was prepared as instructed, and the cell sample was added accordingly. A blank sample, omitting the enzyme mix in the reaction mix, was included. The reaction was incubated for 10–30 min at room temperature while protecting the plate from light. Finally, the absorbance was measured at 570 nm using a Victor Multilabel Plate Reader (Perkin Elmer, Milan, Italy). The data were represented as relative hem content (arbitrary units).

For PPIX synthesis measurement, the method described by Novak and colleagues was followed [21]. All conditions were analyzed in triplicates. In brief, 2 × 10^5^ cells per well were treated with 20 µL of lysis buffer (comprising 125 mM Tris/HCl, pH 7.8; 2 mM DTT; 2 mM EDTA; 10% (*v*/*v*) glycerol; 1% (*v*/*v*) Triton X-100; all from Sigma Aldrich (St. Louis, MO, USA) and incubated for 30 min with gentle shaking. Cell lysis was visually confirmed under a microscope. Once complete lysis was achieved, 80 µL of PPIX extraction solution (consisting of 50% methanol, 35.7% distilled water, 14.3% perchloric acid pre-dissolved to 70% in water, all *v*/*v*) was added, and the cells were shaken for an additional 45 min. Simultaneously, the protein content of the lysed samples was determined using the Bradford assay (Bio-Rad, Hercules, CA, USA). Subsequently, cell extracts were transferred to a black-walled 96-well plate for PPIX fluorescence measurement. The buffer used was previously confirmed not to interfere with PPIX fluorescence measurements. PPIX fluorescence was assessed using a Victor Multilabel Plate Reader (Perkin Elmer, Milan, Italy) with a 405 nm excitation and a 620 nm emission filter, with an integrated readout period of 10 sec per well. Background fluorescence was subtracted by measuring a mixture of 20 µL of lysis buffer and 80 µL of extraction buffer. The obtained data were normalized to the protein content (mg) per sample and presented as relative PPIX content units (arbitrary units). For the quantification of heme, a commercial hemin (oxidized heme) colorimetric assay from Sigma Aldrich (St. Louis, MO, USA; cat. MAK036) was utilized, following the manufacturer’s guidelines and as previously published [12]. All samples were analyzed in triplicates. Initially, cells were homogenized rapidly in 4 volumes of cold hemin assay buffer, followed by centrifugation at 13,000× *g* for 10 min at 4 °C to remove insoluble material. The resulting supernatant was diluted 1000-fold with hemin assay buffer. The reaction mix was prepared as instructed, and the cell sample was added accordingly. A blank sample, omitting the enzyme mix in the reaction mix, was included. The reaction was incubated for 10–30 min at room temperature while protecting the plate from light. Finally, the absorbance was measured at 570 nm using a Victor Multilabel Plate Reader (Perkin Elmer, Milan, Italy). The data were represented as relative hem content (arbitrary units).

### 4.7. Flow Cytometry Analysis

A total of 1 × 10^6^ cells were collected through centrifugation and washed with phosphate-buffered saline (PBS). For mitochondrial ROS analysis [46], cells were suspended in 1 mL of PBS and treated with 5 µM DHR dissolved in dimethyl sulfoxide (DMSO) in the dark for 30 min at 37 °C. After washing with Hank’s Balanced Salt Solution (HBSS), cells were resuspended in 1 mL of HBSS and analyzed using the Attune NxT Flow Cytometer (Life Technologies, Thermo Fisher Scientific, Waltham, MA, USA). For total cellular ROS analysis, cells were resuspended in 1 mL of PBS and treated with 5 µM DCF-DA dissolved in DMSO in the dark for 30 min at 37 °C. After washing with HBSS, cells were resuspended in 1 mL of HBSS and analyzed using the Attune NxT Flow Cytometer (Life Technologies, Thermo Fisher Scientific, Waltham, MA, USA). To distinguish between living and dead cells, propidium iodide (PI) was used as a second indicator dye. Cell cultures were washed with HBSS and resuspended in 1 mL of PI staining solution (0.050 mM PI; TRIS 0.05 M; MgCl_2_ 15 mM, pH 7.7) for analysis. The mitochondrial iron content was investigated using Mito-FerroGreen (Dojindo Laboratories, Dojindo Europe GmbH, Munich, Germany) as the fluorescent probe [17]. Briefly, cells were washed with serum-free RPMI. Mito-FerroGreen was freshly dissolved in DMSO and added to cells at a final concentration of 5 µM. Cells were incubated with the reagent for 30 min at 37 °C in the dark. Following incubation, cells were rinsed and suspended in HBSS for subsequent analysis. For the assessment of the PLP content, the AcRAB6 fluorescent probe was employed [22]. A concentration of 10 µM AcRAB6 were added to the cell culture and incubated for 12 h, following the protocol [22]. Then, cells were washed twice, resuspended in HBSS, and analyzed utilizing the BL1 channel excited by the 488 nm laser. The fluorescence intensities of the histograms were measured based on the geometric mean of the fluorescence intensity. The acquisition was performed on a total of 10,000 events.

### 4.8. Epifluorescence PLP Content Analysis

Epifluorescence analysis was employed to assess the PLP content within the cells [22]. Initially, cells were seeded on a poly-lysine (0.01%)-coated glass coverslips (3 × 10^4^ cells/coverslip) and allowed to adhere for 16 h (60% confluence) before treatment with 10 µM AcRAB6 and 50 µM each of PN, PL, and PM, as reported [22]. After the incubation at 37 °C for 12 h, cells were washed twice with HBSS and treated with 25 nM Mitotracker Red (Thermo Fisher Scientific, Waltham, MA, USA, cat. M7512) at 37 °C for 30 min. Then, cells were again washed with HBSS and analyzed using a Zeiss Axiovert 200 inverted epifluorescence microscope equipped with a 63×/1.30 Ph3 oil objective and appropriate filter sets for the acquisition of AcRAB6 (excitation bandpass 450–490, emission bandpass 515–565) and Mitotracker Red (excitation bandpass 546/12 and emission long pass 590) signals. Images were acquired with a CoolSNAP HQ CCD camera (Roper Scientific, Trenton, NJ, USA), using MetaFluor 6.1 software (Universal Imaging Corporation, Downington, PA, USA) and processed using ImageJ software (ImageJ 1.54f, National Institutes of Health, USA, website: http://imagej.org).

### 4.9. High-Resolution Respirometry

Cell respiration was assessed at 30 °C using the Oxygraph-2k system (Oroboros, Innsbruck, Austria), equipped with two chambers operating at a stirrer speed of 750 rpm. Data acquisition and analysis were performed using DatLab software, version 7.4.0.4. To measure the state III respiration, intact cells were added to the MIR05 buffer in one chamber [19]. Subsequently, cell membranes were permeabilized using an optimal digitonin concentration determined in preliminary experiments (18 µg/mL for 10^6^ cells/mL), following the methods described in previous studies [19,20]. Then, the following complex I + II substrates were added to the chamber at final concentrations: malate (2 mM), pyruvate (5 mM), ADP (2.5 mM), glutamate (5 mM), and succinate (5 mM).

### 4.10. Statistics

Statistical significance was determined using Prism (Graph-Pad) software (version 9.3.1, GraphPad Software, LLC, www.graphpad.com). The statistical analysis of means was conducted using a two-way ANOVA test for the oxidative stress experiments. The two-tailed unpaired Student’s *t*-test was employed for the analysis of all other experiments. The *p* values < 0.05 were considered statistically significant; *p* < 0.05, <0.01 and *p* < 0.001 were marked with *, ** and ***, respectively.

## Data Availability

The authors will make their original data available to future researchers upon a request directed to the corresponding authors.

## Supplementary Materials

The following supporting information can be downloaded at: https://www.mdpi.com/article/10.3390/ijms252413314/s1.
