# Supplementary material for: P2 Receptor Antagonists Rescue Defective Heme Content in an In Vitro SLC25A38-Associated Congenital Sideroblastic Anemia Cell Model
Source: Int J Mol Sci. 2024 Dec 12;25(24):13314. doi: 10.3390/ijms252413314 (PMC11679772; doi:10.3390/ijms252413314)
Supplement: Supplementary file 1 [file ijms-25-13314-s001.zip › ijms-3293892-supplementary.pdf]

Supplementary Table S1

Primer name	Sequence
CB-9571.2_GCD1_F	CCCCTTCTACAGAGTTCCTCCG
CB-9571.2_GCD1_R	CCCTGCCCCCTTCTCAATTC
CB-9571.2 IVT gRNA	CACCGTGTCTCCGACATCTT

List of primers used for the CRISPR/Cas9 editing of the SLC25A38 gene. A certified gRNA (CB-9571.2 IVT gRNA) was designed by a customized approach at Thermo Fisher Scientific on the SLC25A38 gene sequence and was used in transfection experiments of K562 cells, as described in the Materials and Methods section. The validation of off-target effects was tested in house by using the Blat tool of the University of California Santa Cruz (UCSC) Genome Browser on Human (GRCh38/hg38 release), which showed, as a BLAT search result of the CB-9571.2 IVT gRNA sequence, a single match at the map position chr3:39,383,762- 39,383,781 (3p22.1), corresponding to the genomic region of the SLC25A38 exon 1.

Supplementary Figure S1

A

	M	I	Q	N	S	R	P	S	L	L	Q	P	Q	D	V	G	D	T	V...
**SLC25A38**	ATG	ATT	CAG	AAC	TCA	CGT	CCG	TCG	CTG	CTG	CAA	CCC	CAA	GAT	GTC	GGA	GAC	ACG	GTG...
	M	I	Q	N	S	R	P	S	L	L	Q	P	Q				**H**	**G**	**G...**
**A38-low**	ATG	ATT	CAG	AAC	TCA	CGT	CCG	TCG	CTG	CTG	CAA	CCC	CAA	---	---	---	--**CAC**	**GGT**	**GGA...**

B

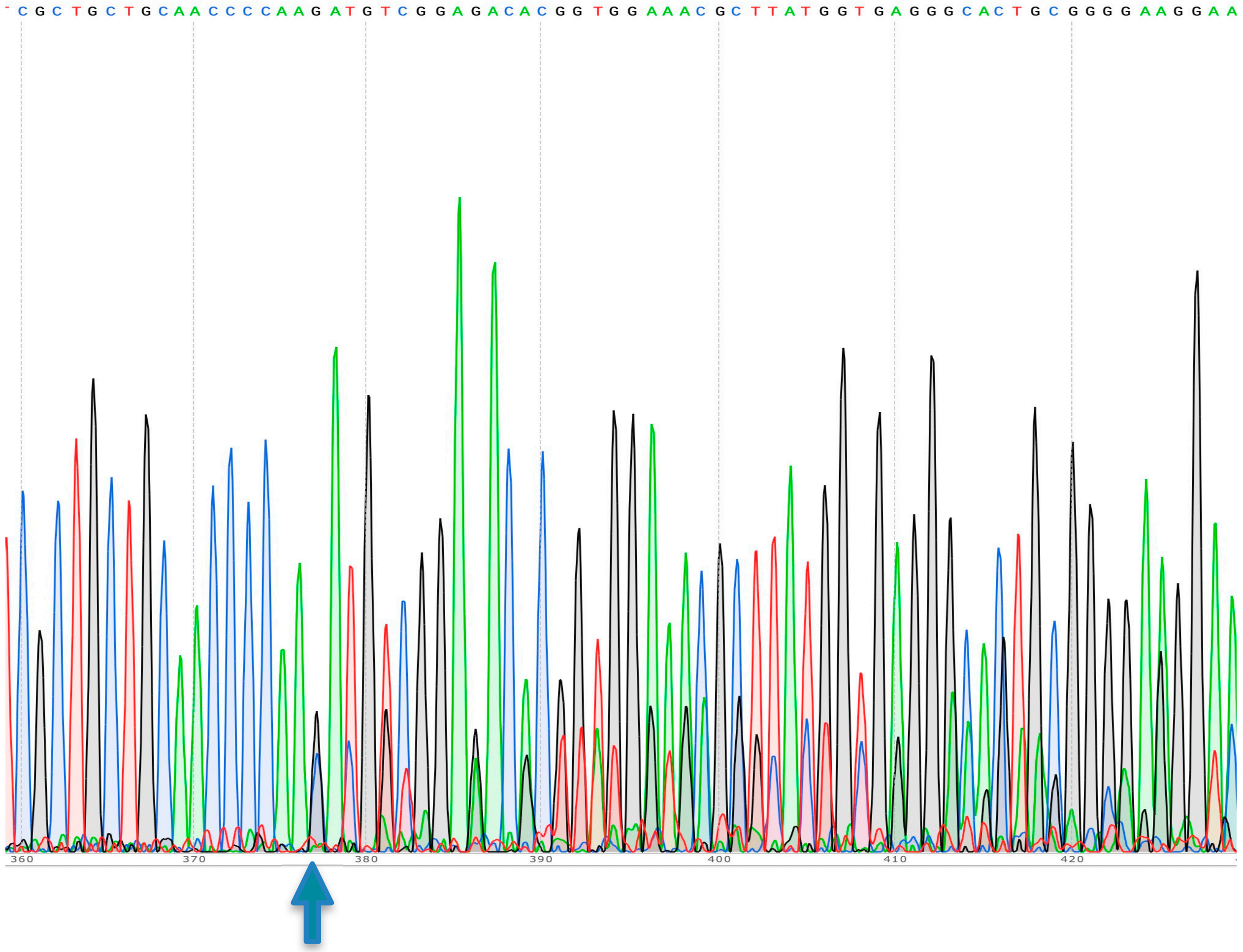

C

MIQNSRPSLLQPQ**HGGNAYVTSGDQGFPVWLHQWDLLYPPFPTSGSP***

Supplementary Fig. S1. Analysis of the SLC25A38 CRISPR/Cas9 induced mutation in A38-low cell line. (A) Sequence alignment between the SLC25A38 wild type gene sequence and the related mutated sequence in the A38-low clone. The deleted DNA sequence is underlined, and the mutated sequence in A38-low is in bold. (B) Electropherogram of the SLC25A38 gene in A38-low genomic DNA. The arrow indicates the heterozygous deletion site. (C) Truncated SLC25A38 peptide. The mutated amino acid sequence is in bold.

Supplementary Figure S2

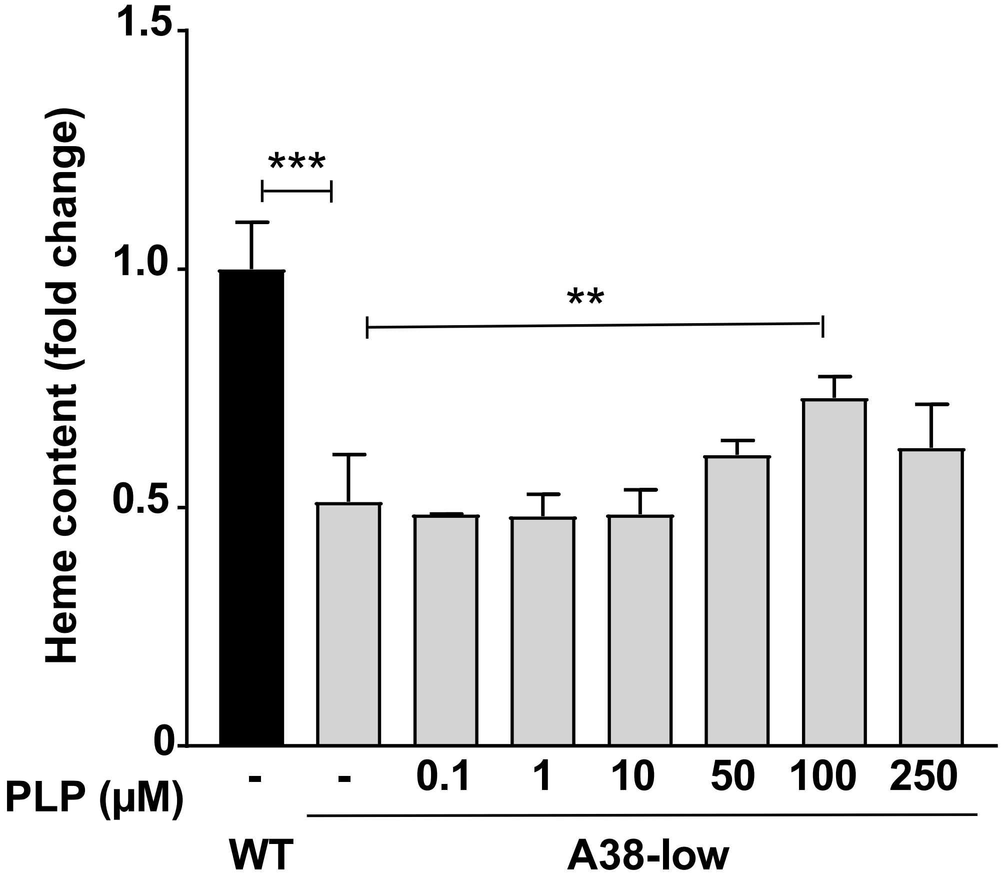

Supplementary Figure S2. Effect of PLP treatment on heme content in A38-low cells. Several concentrations of PLP were evaluated on A38-low cells and heme assay was carried out. The values were related to the WT-untreated sample. Data were expressed as fold changes related to the WT-untreated sample, and represent mean ± SD (** $p \leq 0.01$; *** $p \leq 0.001$; Student's t-test).

Supplementary Figure S3

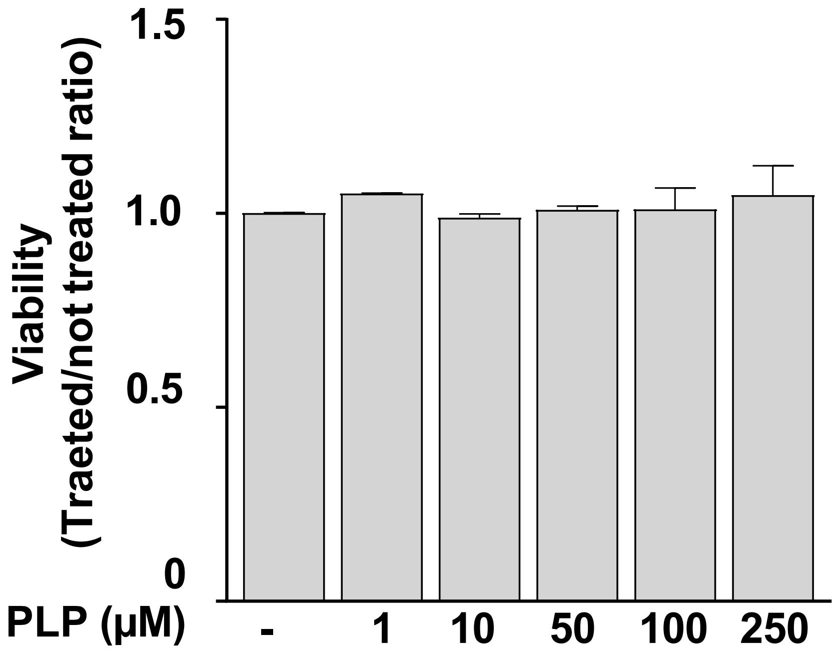

Supplementary Figure S3. Viability of A38-low cells. A38-low cells were treated for 48 h with different PLP concentrations. Data are expressed as mean ± SD (Student's t-test, n=3).

Supplementary Figure S4

A

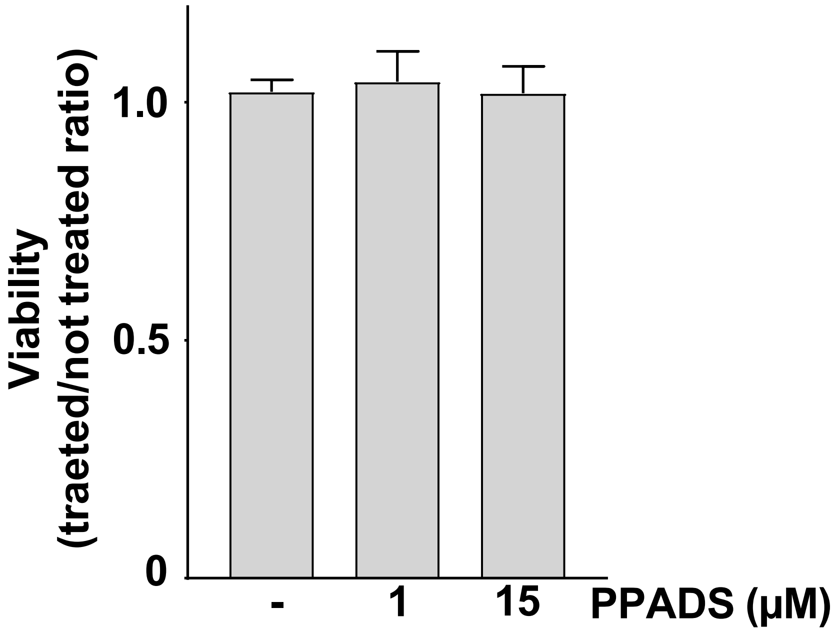

B

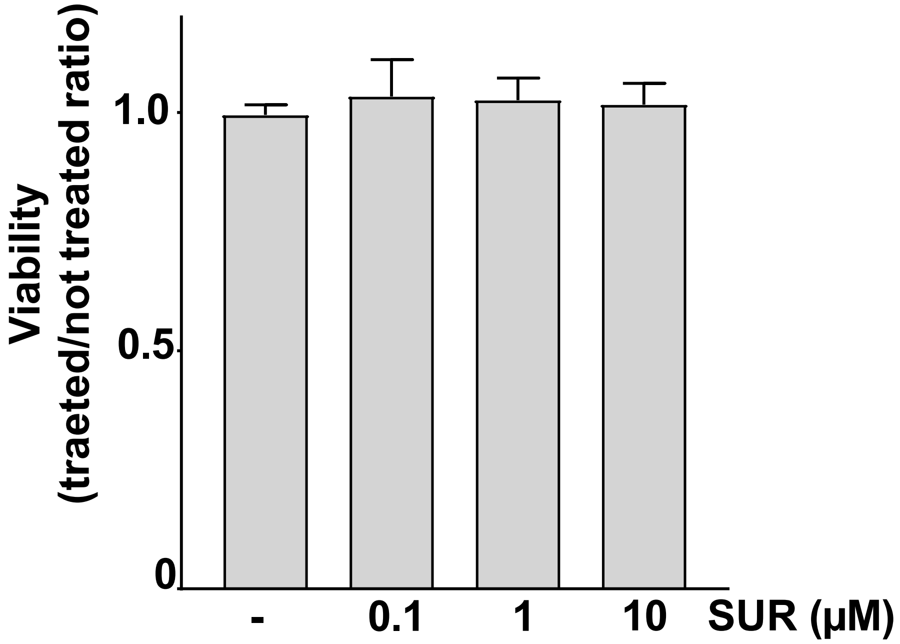

Supplementary Figure S4. Viability of A38-low cells. Cells were treated for 48 h with 1 and 15 µM PPADS (A) and with 0.1, 1 and 10 µM suramin (SUR) (B). Data are expressed as mean ± SD (Student's t-test, n=3).
